# Ecological and Control Techniques for Sand Flies (Diptera: Psychodidae) Associated with Rodent Reservoirs of Leishmaniasis

**DOI:** 10.1371/journal.pntd.0002434

**Published:** 2013-09-12

**Authors:** Thomas M. Mascari, Hanafi A. Hanafi, Ryan E. Jackson, Souâd Ouahabi, Btissam Ameur, Chafika Faraj, Peter J. Obenauer, Joseph W. Diclaro, Lane D. Foil

**Affiliations:** 1 Department of Entomology, Louisiana State University Agricultural Center, Baton Rouge, Louisiana, United States of America; 2 United States Naval Medical Research Unit No. 3, Cairo, Egypt; 3 Syngenta Crop Protection, Cleveland, Mississippi, United States of America; 4 Medical Entomology Laboratory, National Institute of Hygiene, Rabat, Morocco; 5 Department of Epidemiology and Disease Control, Ministry of Health, Rabat, Morocco; The Faculty of Medicine, The Hebrew University of Jerusalem, Israel

## Abstract

**Background:**

Leishmaniasis remains a global health problem because of the substantial holes that remain in our understanding of sand fly ecology and the failure of traditional vector control methods. The specific larval food source is unknown for all but a few sand fly species, and this is particularly true for the vectors of *Leishmania* parasites. We provide methods and materials that could be used to understand, and ultimately break, the transmission cycle of zoonotic cutaneous leishmaniasis.

**Methods and Findings:**

We demonstrated in laboratory studies that analysis of the stable carbon and nitrogen isotopes found naturally in plant and animal tissues was highly effective for linking adult sand flies with their larval diet, without having to locate or capture the sand fly larvae themselves. In a field trial, we also demonstrated using this technique that half of captured adult sand flies had fed as larvae on rodent feces. Through the identification of rodent feces as a sand fly larval habitat, we now know that rodent baits containing insecticides that have been shown in previous studies to pass into the rodents' feces and kill sand fly larvae also could play a future role in sand fly control. In a second study we showed that rubidium incorporated into rodent baits could be used to demonstrate the level of bloodfeeding by sand flies on baited rodents, and that the elimination of sand flies that feed on rodents can be achieved using baits containing an insecticide that circulates in the blood of baited rodents.

**Conclusions:**

Combined, the techniques described could help to identify larval food sources of other important vectors of the protozoa that cause visceral or dermal leishmaniasis. Unveiling aspects of the life cycles of sand flies that could be targeted with insecticides would guide future sand fly control programs for prevention of leishmaniasis.

## Author Summary

The sand fly *Phlebotomus papatasi* is the vector of *Leishmania major*, which is a causative agent of zoonotic cutaneous leishmaniasis in the Old World. Despite its impact on public health, leishmaniasis remains a problem because of the substantial holes that remain in our understanding of sand fly ecology and the failure of traditional vector control methods. We conducted laboratory and field trials to demonstrate for the first time that sand flies are feeding on the feces of rodents (the reservoirs of *L. major*). Based on this new information, we now can identify areas where sand flies feed on rodent feces and therefore could be targeted using rodent baits containing an insecticide that passes into the feces of baited rodents. We also conducted a field trial using rodent bait containing an insecticide that circulates in the blood of baited rodents. We demonstrated that sand flies feeding on baited rodents were killed, and confirmed this finding using a tracer system that marks sand flies that have fed on baited rodents. Combined, these results show that we now have methods and materials that could be used to break the transmission cycle of zoonotic cutaneous leishmaniasis and target sand flies closely associated with rodents.

## Introduction

Phlebotomine sand flies are vectors of viruses, bacteria, and protozoa that can cause disease in man. Most importantly, sand flies are vectors of *Leishmania* parasites, which can cause disfiguring dermal lesions or life-threatening visceral disease. The World Health Organization (WHO) considers leishmaniasis to be an emerging and uncontrolled disease that disproportionately affects the poor [1]. Leishmaniasis remains a significant global health problem in part because traditional vector control methods recommended by WHO for sand fly control are not effective in prevention of transmission. Insecticide applications targeting adult sand flies and personal protective measures against sand fly bites, such as bed nets, insect repellents, and insecticide-treated clothing, do not appear to be effective and are often not available to or practical for at-risk populations in low- and medium-income countries [2], [3]. One reason that effective sand fly control methods are not available is that our understanding of key aspects of sand fly ecology is so limited that designing methods of control is not possible. Larval control is a vital component of most vector control programs to reduce populations of medically important insects. However, very little is known about the larval ecology of most species of sand flies due to the difficulty of finding specimens in nature [4]. In order to determine the future potential for control of sand flies, there is a need to obtain basic knowledge about the ecology of immature sand flies.

*Leishmania major* is a causative agent of zoonotic cutaneous leishmaniasis (ZCL) in the Old World, where it is circulated among populations of burrow-dwelling rodents through the bite of infectious female sand flies. Human infections occur when people encroach on this enzootic cycle. *Phlebotomus papatasi* is the principal vector of *L. major* in North Africa, the Middle East, and Asia. Purported larval habitats of *P. papatasi* include rodent burrows, open ground, domestic animal shelters, human habitation, and stone piles, but the larval food source likely is the most important factor that determines the suitability of potential larval habitats for *P. papatasi* in ZCL foci [4].

One objective of this study was to evaluate the use of a technique to link adult sand flies to their larval food sources. Since sand fly larvae cannot be sampled reliably, the importance of putative larval habitats currently cannot be quantified in terms of their actual contribution to the overall adult population of sand flies in an area. However, indirect methods have been used to identify the diets of larvae of other insects through comparison of the naturally occurring stable carbon and nitrogen isotopes found in different larval diets and adult insects. This technique previously has been used with agricultural insect pests to determine the larval host plants for fall armyworms (*Spodoptera frugiperda*) and cotton bollworms (*Helicoverpa zea*) through a comparison of larval host plant tissue and the tissue of adult moths [5], [6].

The second objective of this study was to capitalize on the fact that rodents are the only natural reservoirs for *L. major* and use an ivermectin-treated rodent bait to kill adult female sand flies that take bloodmeals from baited rodents. Ivermectin (IVM) acts as a systemic insecticide that kills adult female sand flies that feed on rodents before the sand flies become infectious vectors and take subsequent bloodmeals, a technique which ultimately could be used to break the cycle of transmission of *L. major* [7]. We also have incorporated the trace element rubidium (Rb) and the fluorescent dye rhodamine B (RhB) into rodent baits, which can be detected in female sand flies that have taken a bloodmeal from a baited rodent [8], [9]. A small-plot field experiment in Morocco was conducted to determine whether rodent baits containing Rb could be used to demonstrate the level of bloodfeeding by sand flies on baited rodents and to demonstrate whether rodent baits containing IVM could be used to eliminate sand flies that feed on these rodents.

## Materials and Methods

### Ethics statement

This study was performed in strict accordance with the recommendations in the Guide for the Care and Use of Laboratory Animals of the National Institutes of Health. The protocols were approved by the Institutional Animal Care and Use Committee of Louisiana State University (Permit Number: 11-035).

### Stable isotope analysis - Laboratory proof of concept

Three laboratory experiments were conducted to demonstrate proof of concept for the use of stable carbon and nitrogen isotope analysis to establish an association between adult sand flies and their larval food sources. In these experiments samples of animal food or feces (0.5–1.0 mg), or a single adult sand fly was placed into a 5×9 mm tin capsule. The tin capsule was compressed tightly around the sample using sterilized forceps, and the tissue within each tin capsule was converted to CO_2_ by micro-Dumas combustion using a Costech ECS4010 Elemental Analyzer coupled to a Thermo Finnigan Delta plus Advantage Mass Spectrometer using a Conflo II Interface. The stable isotopic compositions of carbon and nitrogen in the samples were obtained and reported as δC or δN (values in parts per thousand relative to a standard of known composition). Carbon stable isotope values (δ^13^C) were ratios of stable isotopes ^13^C:^12^C and were reported relative to the Vienna Pee Dee Belemnite (V-PDB) scale. Nitrogen stable isotope values (δ^15^N) were ratios of stable isotopes ^15^N:^14^N and were reported relative to atmospheric N_2_.

In the first experiment, rabbits (*Oryctolagus cuniculus*) were fed LabDiet 5321 (PMI Nutrition International, Brentwood, MO), and hamsters (*Mesocricetus auratus*) were fed LabDiet 5001 (PMI Nutrition International, Brentwood, MO). Samples of the diets and the feces of rabbits and hamsters were collected, air-dried at room temperature overnight, and stored at −80°C. Portions of the feces of rabbits and hamsters that had been collected were fed to sand fly larvae, and the remaining feces were retained for analysis. The sand flies used in these experiments were from a laboratory colony of a Turkish strain of *P. papatasi* that had been maintained at the LSU AgCenter (Baton Rouge, LA, USA) since 2005. Sand fly rearing containers were 120 mL plastic specimen cups with a plaster of Paris base. Sand fly eggs were transferred onto the plaster surface of the containers, and, upon hatching, sand fly larvae were fed the feces of rabbits or hamsters *ad libitum*. Sand fly larvae were maintained in darkness at 28°C and 90% relative humidity. Adult sand flies that emerged were killed by freezing and stored in 100% ethanol.

Six groups of samples were evaluated: rabbit chow, rabbit feces, sand flies reared on rabbit feces, hamster chow, hamster feces, and sand flies reared on hamster feces. A total of 27–30 samples were analyzed for each treatment, except for sand flies reared on rabbit feces, where 14 samples were analyzed (13 other samples were inadvertently destroyed prior to analysis). A hierarchical cluster analysis (PROC CLUSTER) was used to place samples into groups based upon similar δ^13^C and δ^15^N values [10]. Mean δ^13^C and δ^15^N values (the dependent variables) were compared by Wilks' lambda multivariate analysis of variance (MANOVA) using PROC GLM [10]. Discriminant analysis (PROC DISCRIM) was used to predict whether adult sand flies had been reared on rabbit or hamster feces and to measure the accuracy of these predictions [10].

The second experiment was conducted to determine the influence of sand fly sex and sugar feeding on results from stable isotope analysis. Sand fly larvae were reared on a standard laboratory larval diet [11]. As sand flies emerged as adults, they were segregated by sex and transferred into 250 mL glass jars. Sand flies either were allowed to feed from a cotton ball saturated with 20% sucrose solution or were starved. After 48 h, all sand flies were killed. The abdomens of sand flies that had been provided with sugar solution were examined, and only sand flies that had taken a sugar-meal were included in the analysis for this treatment. Four groups were evaluated: male sugar-fed, male unfed, female sugar-fed, and female unfed sand flies. A total of 10–13 samples per treatment were analyzed. Mean δ^13^C and δ^15^N values (the dependent variables) were compared (P<0.05) by Wilks' lambda MANOVA using PROC GLM [10].

The third laboratory experiment was conducted to determine the influence of blood meals taken by female sand flies on δ^13^C and δ^15^N values. Adult female sand flies that had been reared as larvae on standard laboratory diet were allowed to take a bloodmeal from a hamster that had been immobilized using an intraperitoneal injection of ketamine HCl (100 mg/kg body weight) plus xylazine HCl (10 mg/kg body weight). Immediately after feeding, fully engorged sand flies were killed by freezing. Female sand flies that had emerged from the same cohort of larvae but had not taken a bloodmeal also were killed. Two groups were evaluated: female sand flies that had taken a bloodmeal and unfed female sand flies. A total of 25 samples were analyzed for each treatment. Mean δ^13^C and δ^15^N values (the dependent variables) were compared (P<0.05) by Wilks' lambda MANOVA using PROC GLM [10].

### Stable isotope analysis – Field trial

Sand flies were captured using light traps baited with dry ice (Model 1012, John W Hock, Co., Gainesville, FL) in Oued El Biiaza, Figuig Province, Morocco (lat 32.50, long 01.79) in September 2011. The sand fly collection site was 2 m away from a cluster of active burrows of Shaw's jirds (*Meriones shawi*) and was more than 1 km away from human habitation or livestock pens. Vegetation in the study areas was limited to thorn bushes, small shrubs, and small patches of grass. Samples of feces of jirds collected outside of the entrances to burrows and the plant (*Salsola* sp.) fed on by jirds also were collected. Jirds were observed feeding on the leaves and stems of *Salsola* sp. plants, and cut portions of the plant were observed at the entrances to jird burrows. Fecal and plant samples were stored frozen at −80°C. Sand flies were identified by the morphology of reproductive structures in terminal segments of the abdomen and stored in 100% ethanol [12]. Only sand flies identified as *P. papatasi* were analyzed.

In order to obtain reference δ^13^C and δ^15^N values for sand flies known to have fed as larvae on the feces of jirds collected from Oued El Biiaza, a portion of the jird feces collected in the field was fed to larvae of *P. papatasi* in the laboratory as described above. Adult sand flies that emerged were killed by freezing and stored in 100% ethanol.

Four groups of samples were analyzed to obtain δ^13^C and δ^15^N values: sand flies collected near the jird burrows (a total of 22 adult females of *P. papatasi*), jird feces (a total of 30 samples: 3 samples from 10 fecal pellets), tissue of the *Salsola* sp. plant (a total of 9 samples), and sand flies reared on field-collected jird feces in the laboratory (a total of 9 adult females of *P. papatasi*).

Field-collected sand flies were segregated into groups based on similar δ^13^C and δ^15^N values using hierarchical cluster analysis with Ward's minimum variance linkage [10]. Subsequently, Wilks' lambda MANOVA was conducted to determine whether these clusters defined groups with significantly different δ^13^C and δ^15^N values (the dependent variables) and to determine whether these clusters were significantly different from the δ^13^C and δ^15^N values of sand flies that had been reared in the laboratory on field-collected jird feces, field-collected jird feces, or the plant tissue fed on by jirds in the field [10].

### Control of bloodfeeding sand flies using a systemic insecticide

A 2-week long experiment was conducted in Oued El Biiaza. Six study sites were selected, each centered on a cluster of active jird burrows beneath thorn bushes. Half of the study sites were treated with rodent bait (dry pellets made from alfalfa and spinach) containing 5000 mg/kg rhodamine B (RhB; Sigma, St. Louis, MO) and 1000 mg/kg Rubidium chloride (Sigma, St. Louis, MO), which was equivalent to 706.9 mg/kg Rb. The other sites were treated with rodent baits containing 5000 mg/kg RhB, 706.9 mg/kg Rb, and 20 mg/kg IVM (Sigma, St. Louis, MO). Approximately 250 g of bait was placed directly on the ground under the bushes covering active burrows of jirds at each site. Sites were baited every 4 d from 20 September–2 October 2011. When sites were re-baited, samples of rodent feces were collected from each site and stored frozen for detection of RhB in order to confirm that rodents were feeding on baits throughout the experiment. Feces were examined under fluorescence microscopy for the presence of RhB using methods described by Mascari and Foil [8].

The sand fly population at each site was sampled using light traps baited with dry ice every other day during the study period. One trap was run overnight at each site, and all captured sand flies were killed and stored in dry ice for up to 12 d. The sand flies were kept in a cold chain and transported to Louisiana State University (Baton Rouge, LA, USA). The sand flies first were examined for the presence of Rb using a hand-held X-ray fluorescence analyzer, and then cleared and identified [9]. The number of females of *P. papatasi* collected and the proportion of females of *P. papatasi* that were positive for Rb were determined for each site. The numbers of adult females of *P. papatasi* that were collected at each treatment were compared using Student's t-test [10].

We also conducted a laboratory experiment to confirm efficacy of the baits used in the field study. Bait containing RhB+Rb+IVM or RhB+Rb, or untreated bait (dry pellets made from alfalfa and spinach) was fed to captive jirds (*Meriones unguiculatus*) in the lab. Three jirds were assigned to each of the 3 diet groups, housed individually, and provided with 20 g of bait for a 24 h period. After feeding on bait for 24 h, the jirds were provided with untreated LabDiet 5001 (PMI Nutrition International, Brentwood, MO). Each jird then was chemically immobilized and placed individually into a 3.8 L clear plastic cage with 25 female sand flies (*P. papatasi*) aged 3–5 d. Sand flies were allowed to feed on a jird for at least 30 minutes before the jird was removed and recovered. Sand flies then were provided with 20% sucrose solution *ad libitum*. Subsequently, another cohort of 3–5 d old female sand flies was bloodfed on the hamsters 7 d after the jirds had been fed bait. In each bioassay, the mortality of sand flies was assessed after 48 h, and the number of bloodfed sand flies that were alive and dead was recorded. The percent mortality of sand flies that had taken bloodmeals from hamsters in each diet group was compared using ANOVA performed with the GLM procedure, and significantly different means were separated using the Tukey multiple comparison procedure [10]. Bloodfed sand flies (alive or dead) also were analyzed for the presence of Rb.

## Results

### Stable isotope analysis - Laboratory proof of concept

**Figure 1:**
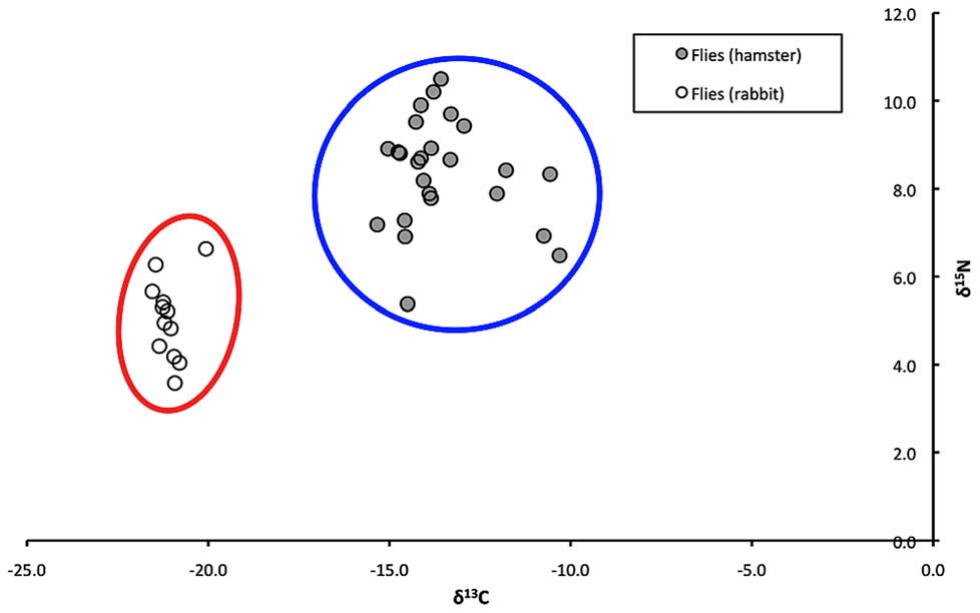
Stable carbon and nitrogen isotope ratios (δ^13^C and δ^15^N) of individual adult sand flies that had been reared in the laboratory on the feces of either hamsters or rabbits. Hierarchical cluster analysis identified 3 significantly different groups of samples based on δ^13^C and δ^15^N values. The cluster circled in red contained all sand flies reared as larvae on rabbit feces, and the cluster circled in blue contained all sand flies reared as larvae on hamster feces.

In the first laboratory experiment, stable carbon and nitrogen isotope analysis was conducted to obtain δ^13^C and δ^15^N values for the food and feces of rabbits and hamsters, and adult sand flies that had been reared as larvae on the feces of these animals. Hierarchical cluster analysis placed samples into two clusters defined by similar δ^13^C and δ^15^N values: “rabbit cluster” (sand flies reared as larvae on the feces of rabbits, rabbit feces, and rabbit food) and “hamster cluster” (sand flies reared as larvae on the feces of hamsters, hamster feces, and hamster food; Fig. 1). There were overall significant differences between the δ^13^C and δ^15^N values of sand flies, feces, and food (Wilks' lambda = 0.01715; *F* = 197.746; df = 10, 298; *P*<0.0001). However, the δ^13^C and δ^15^N values of samples within the “rabbit cluster” were not significantly different from each other (P>0.05); the δ^13^C and δ^15^N values of samples within the “hamster cluster” also were not significantly different from each other (P>0.05). Discriminant analysis showed that categorizing samples in either the “rabbit cluster” or the “hamster cluster” based upon δ^13^C and δ^15^N values was 100% accurate. In the second laboratory experiment, we evaluated the potential influence of the sex of sand flies or sugar feeding on δ^13^C values or δ^15^N values of adult sand flies and determined that there were no significant differences between male or female sand flies, or sand flies that either had ingested sugar or not (Wilks' lambda = 0.75080; *F* = 2.054; df = 6, 80; *P* = 0.068). In the third laboratory experiment, we evaluated the potential influence of bloodfeeding on δ^13^C values or δ^15^N values of adult female sand flies and determined that there were no significant differences between sand flies that had taken a bloodmeal or were unfed (Wilks' lambda = 0.92771; *F* = 1.831; df = 2, 47; *P* = 0.171).

### Stable isotope analysis – Field trial

**Figure 2:**
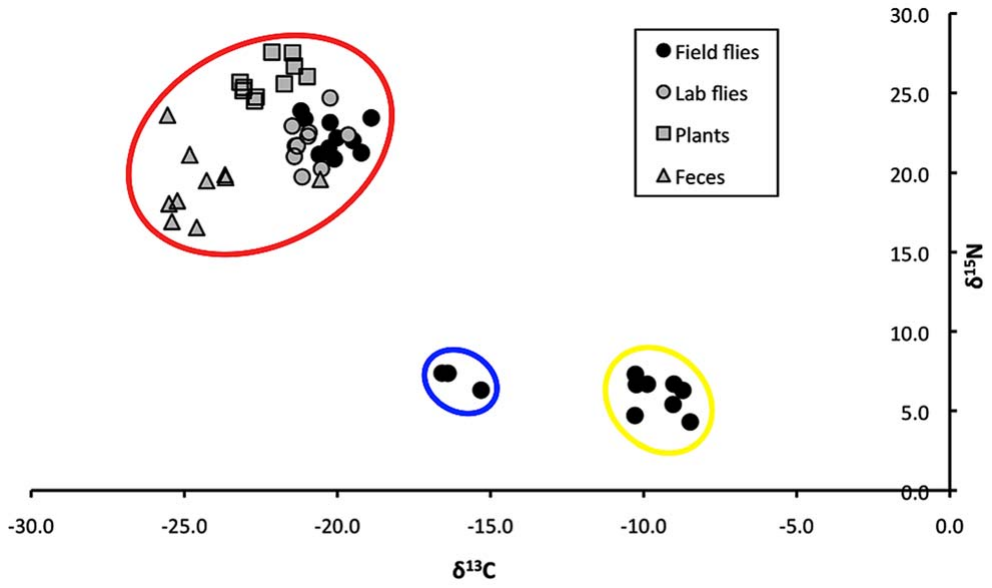
Stable carbon and nitrogen isotope ratios (δ^13^C and δ^15^N) of feces of wild jirds, tissue of the plant fed on by jirds, and individual wild adult sand flies that had been collected near jird burrows or reared in the laboratory as larvae on field-collected jird feces. Hierarchical cluster analysis identified 3 significantly different groups of samples based on δ^13^C and δ^15^N values. The cluster circled in red contained feces of wild jirds, tissue of the plant fed on by jirds, individual sand flies reared in the laboratory as larvae on field-collected jird feces, and 50.0% of the sand flies captured near the jird burrows. The clusters circled in blue and yellow contained 36.4% and 13.6% of the sand flies captured near the jird burrows, respectively.

**Figure 3:**
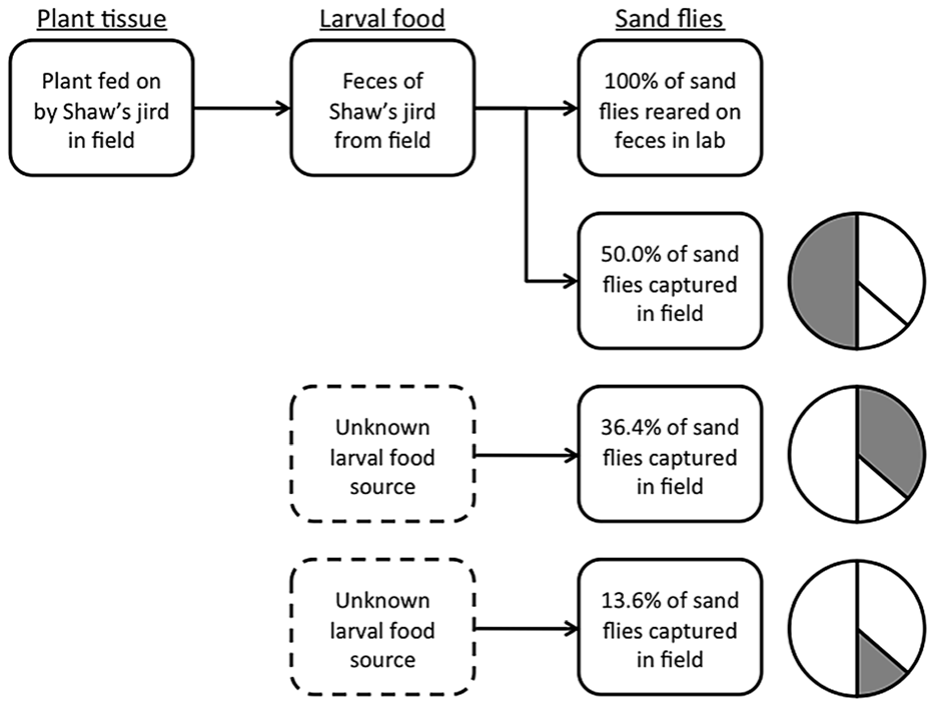
Diagram of the use of stable isotope analysis to link the plants used for forage by jirds to the feces produced by these jirds and to sand flies that had fed on these feces as larvae. The δ^13^C and δ^15^N values of 100.0% of sand flies fed jird feces as larvae in the lab and 50.0% of sand flies captured at the study site matched the field collected jird feces. Stable isotope analysis also indicated the remaining 50.0% of sand flies captured at the study site had fed as larvae on one of two distinct alternative (and currently unknown) food sources other than jird feces.

Stable carbon and nitrogen isotope analysis was conducted to obtain δ^13^C and δ^15^N values for sand flies collected near the burrows of jirds. Hierarchical cluster analysis of adult sand flies produced 3 clusters based upon similarity of δ^13^C and δ^15^N values, which contained 50.0, 36.4, and 13.6% of the sand flies (Fig. 2). The δ^13^C and δ^15^N values of these field-collected sand flies, sand flies from the laboratory colony that had been reared on field-collected jird feces, field-collected jird feces, and the plant tissue eaten by jirds in the field were significantly different (Wilks' lambda = 0.21755; *F* = 142.570; df = 10, 88; *P*<0.0001). However, the δ^13^C and δ^15^N values of field-collected sand flies in the cluster that contained 50.0% of the total sand flies captured, all of sand flies from the laboratory colony that had been reared on field-collected jird feces, all of the field-collected jird feces, and all tissue samples of the plant fed on by jirds in the field were not significantly different from each other (P>0.05; Fig. 3). The other two clusters of field-collected sand flies had significantly different δ^13^C and δ^15^N values from each other and from all other groups of samples (*P*<0.05).

### Control of bloodfeeding sand flies using a systemic insecticide

**Figure 4:**
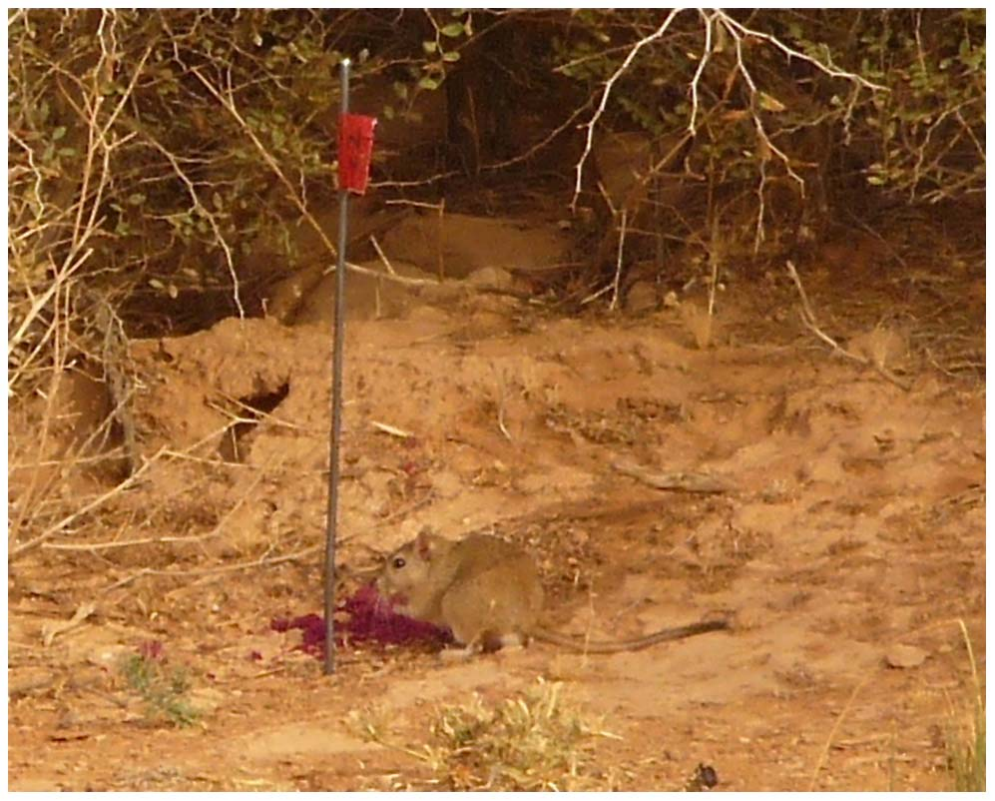
Shaw's jird feeding on bait that had been placed at a study site.

At each study site, all bait had been removed when the sites were re-baited every 4 d. Jirds also were observed feeding on bait at the study sites that had been treated either with baits containing RhB+Rb+IVM or RhB+Rb (Fig. 4). Feces collected at each site were visibly marked pink by RhB and were positive for the presence of RhB when examined using fluorescence microscopy, indicating that the jirds had fed on bait at all sites.

**Figure 5:**
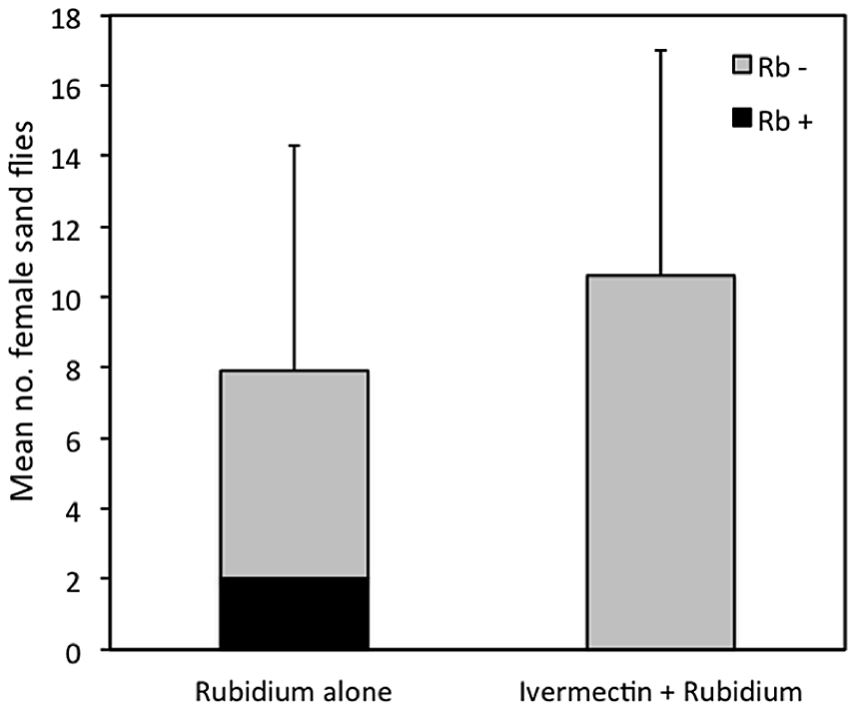
The mean number of females of *P.* *papatasi* captured per night and the mean number of sand flies that was positive for the presence of rubidium at sites treated with rodent bait containing rubidium plus ivermectin or rubidium alone.

At sites treated with rodent baits containing RhB+Rb, an average of 28.3±18.1% of females of *P. papatasi* were positive for the presence of Rb (Fig. 5). None of the sand flies captured at sites treated with rodent baits containing RhB+Rb+IVM were positive for Rb. There was no significant difference between the mean number of females of *P. papatasi* collected at sites treated with baits containing RhB+Rb (7.9±6.4) or RhB+Rb+IVM (10.6±7.2; *t = *0.8289; df = 16; *P = *0.4193). None of the females of *P. papatasi* that were captured throughout this study at any of the field sites were engorged with blood.

In the laboratory experiment in which the efficacy of rodent baits was tested against sand flies, 100% of sand flies died within 48 h after taking a bloodmeal from a jird that had been fed bait containing RhB+Rb+IVM 0 or 7 d after the jird had had eaten the bait. All sand flies that took a bloodmeal from a jird fed bait containing RhB+Rb or an untreated bait 0 or 7 d after the jird had had eaten the bait were alive after 48 h. All sand flies that took a bloodmeal from a jird fed a bait containing RhB+Rb+IVM or RhB+Rb were positive for the presence of Rb, while all sand flies that took a bloodmeal from a jird fed untreated bait were negative for the presence of Rb.

## Discussion

The results of our first series of experiments demonstrated that stable carbon and nitrogen isotope analysis was a highly effective technique for linking adult sand flies with the feces on which they had fed as larvae. In the laboratory we showed experimentally that after mean δ^13^C and δ^15^N values have been obtained for different potential larval diets, individual adult sand flies could be accurately categorized as having been reared on one of these diets. We also showed that the consumption of food by adult sand flies (either sugar or blood) or the sex of sand flies did not significantly alter their δ^13^C and δ^15^N values. In the field component of this study, we demonstrated through analysis of δ^13^C and δ^15^N values of rodent fecal samples and samples of plant tissues collected from the field that jirds were feeding exclusively on one of the plants (*Salsola* sp.) present at the study site, and that 50% of the adults of *P. papatasi* in that area had fed on the feces of jirds as larvae (Fig. 3).

The immediate implication of the results of this component of our study is that larval control now is an option to reduce populations of *P. papatasi* associated with *M. shawi* in Morocco. Historically, larval control has been hindered by our lack of understanding of the larval habitats of sand flies and the failure of insecticide applications made directly into rodent burrows, probably due to the failure of the insecticide to reach larval microhabitats deep within the burrows [13].

Stable isotope analysis provided quantitative evidence linking sand fly larvae to rodent feces for the first time, which could lead to evidence-based larval control of sand flies using a feed-through rodent bait in the future. We and others recently have developed rodent baits containing various insecticides that pass into the feces of rodents and kill larvae that feed on these feces, effectively turning a rodent into a vehicle to deliver an insecticide to the precise sand fly larval habitat [14], [15]. However, because these ecological data previously have not been available, these studies have not been based on concrete knowledge of sand fly larval food sources and habitats.

Foci of *L. major* transmission are ecologically diverse, ranging from areas where there are few possible sand fly larval habitats available other than rodent feces (such as our study site in Morocco), to areas where feces of multiple animal species and other organic matter are abundant. It is possible that larvae of both Old World and New World sand fly vectors do not feed on the feces of rodents in all habitats throughout their distribution, and that alternative larval habitats exist for vector species even in areas where their larvae do feed on rodent feces. At our study site, we discovered that there were two discrete alternative larval food sources present in the area. While currently unknown, we believe that in future studies we will be able to identify these alternative habitats through analysis of additional organic materials that could be used as food sources by larvae, but that had not been collected during this current study. The only apparent larval habitat for sand flies at the site was feces of the Shaw's jird. Therefore, it also is possible that other adult sand flies from different locations had moved into the study area. Information on the movement of sand flies from larval habitats would be important to know in the future in order to determine whether area-wide control using exclusively a rodent bait containing only a larvicide would be effective.

Stable carbon and nitrogen isotope analysis has broad applications for answering very important questions surrounding the ecology of larvae of many different species of sand flies around the world, and could provide information that may lead to identifying new approaches to control. Some obvious examples of other potential applications of this tool would be identifying the larval habitats of vectors of *Leishmania donovani* in India that may be associated with manure in cattle sheds, vectors of *Leishmania tropica* in the Eastern Mediterranean region that may be associated with the feces of rock hyraxes, vectors of *Leishmania infantum* in South America that may be associated with chicken manure, and vectors of *Leishmania mexicana* in southern Texas that may be associated with the feces of woodrats [16]–[19].

The results of the second part of our field study constitute proof of concept for the targeted control of sand flies that take bloodmeals from the rodent reservoirs of *L. major*, and the potential for the interruption of the transmission of *L. major* using applications of systemic insecticide-treated rodent baits. Specifically, we demonstrated bloodfeeding by sand flies on baited jirds using Rb and the elimination of all sand flies that had fed on rodents targeted with IVM-treated bait. The results of this study also demonstrated the utility of Rb as a bio-indicator when used in conjunction with insecticide-treated rodent baits. Previous laboratory studies have shown that Rb is retained in detectable levels by bloodfeeding sand flies, but not in sand flies that fed as larvae on feces containing Rb [9]. Therefore, the only route by which adult female sand flies could have acquired Rb in this study is through bloodfeeding on baited rodents.

By incorporating Rb into baits in this study, it was possible to determine, through the detection of the trace element in female sand flies, the proportion of females of *P. papatasi* that took bloodmeals from baited rodents at each study site over the course of 2 weeks. Because rubidium persists for at least 14 d in sand flies that acquired the element through bloodfeeding, we were able to demonstrate that more than a quarter of captured females of *P. papatasi* had fed on baited rodents at sites not treated with IVM despite the fact that no engorged females were captured at any of our study sites. Furthermore, the absence of Rb-positive sand flies among the sand flies captured at sites treated with rodent baits containing IVM suggests that there were no instances where the insecticide treatment failed to kill sand flies that had previously taken a bloodmeal from a rodent baited with the IVM bait.

Our insecticide trial was conducted at the end of the season of activity for adult sand flies in Morocco, and the two-week study period was designed only to capture the impact of rodent bait containing a systemic insecticide on the survival of bloodfeeding sand flies. This approach ultimately could be used to break the transmission cycle of *L. major* parasites, similar to what Kobylinski et al. described for reducing *Plasmodium* infection rates in malaria vectors through administration of ivermectin to human hosts [20]. However, given the high level of bloodfeeding by sand flies on targeted jirds (as indicated by the presence of Rb in adult females) and the scarcity of alternative bloodmeal hosts in this study area, it is possible that a sand fly population reduction (in addition to interruption of *L. major* transmission) could be achieved if insecticide-treated rodent baits were used throughout the period of adult sand fly activity. A larger scale, 6-month long field study currently is being conducted in Morocco to determine the effects of baits containing IVM on populations of sand fly vectors.

In the absence of a vaccine, control of zoonotic leishmaniasis must rely on suppression of vectors at key points in their life cycle. The methods and materials described in this study could be used in the future to break the transmission cycle of zoonotic leishmaniasis. The results of this current study demonstrated the usefulness of a tool to reveal the larval habitats of sand flies, opening the door to future evidence-based larval control options against sand flies. We also demonstrated that the use of ivermectin-treated rodent baits can eliminate the most epidemiologically important subset of the sand fly populations in *L. major* foci, sand flies that feed on rodent reservoirs, even without overall population control. Combined, these techniques could help in the development of effective sand fly control programs that would lead to the suppression of transmission of *L. major*, and potentially help reduce the burden of this currently uncontrolled and emerging disease. The results of our studies are presented as a challenge to international agencies to consider implementing large-scale trials to attempt suppression of the transmission of zoonotic cutaneous leishmaniasis, which affects the most impoverished people on the planet.
